# Detection and Prevalence Patterns of Group I Coronaviruses in Bats, Northern Germany

**DOI:** 10.3201/eid1404.071439

**Published:** 2008-04

**Authors:** Florian Gloza-Rausch, Anne Ipsen, Antje Seebens, Matthias Göttsche, Marcus Panning, Jan Felix Drexler, Nadine Petersen, Augustina Annan, Klaus Grywna, Marcel Müller, Susanne Pfefferle, Christian Drosten

**Affiliations:** * Centre for Bat Protection and Information, Bad Segeberg, Germany; † University of Kiel, Kiel, Germany; ‡ Bernhard Nocht Institute for Tropical Medicine, Hamburg, Germany; § University of Bonn Medical Centre, Bonn, Germany

**Keywords:** coronavirus, SARS, bats, research

## Abstract

We tested 315 bats from 7 different bat species in northern Germany for coronaviruses by reverse transcription–PCR. The overall prevalence was 9.8%. There were 4 lineages of group I coronaviruses in association with 4 different species of verspertilionid bats (*Myotis dasycneme, M. daubentonii, Pipistrellus nathusii,* *P. pygmaeus*). The lineages formed a monophyletic clade of bat coronaviruses found in northern Germany. The clade of bat coronaviruses have a sister relationship with a clade of Chinese type I coronaviruses that were also associated with the *Myotis* genus (*M. ricketti*). Young age and ongoing lactation, but not sex or existing gravidity, correlated significantly with coronavirus detection. The virus is probably maintained on the population level by amplification and transmission in maternity colonies, rather than being maintained in individual bats.

## Abstract 2

The virus is probably maintained on the population level by amplification and transmission in maternity colonies.

Coronaviruses are enveloped viruses with plus-stranded RNA genomes of 26–32 kb, the largest contiguous RNA genomes in nature (*1*,*2*). They are classified in 3 groups: groups I and II (pathogenic viruses for mammals) and group III (poultry). Group I contains 2 prototypic human pathogenic coronaviruses: human coronavirus (hCoV)-NL63 and hCoV-229E (*3*,*4*). Human pathogenic group II viruses include hCoV-HKU1 and hCoV-OC43 (*5*,*6*). Another human pathogenic coronavirus within a subgroup of group II (termed group IIb) is the severe acute respiratory syndrome (SARS) coronavirus (*7*–*10*). It caused an international epidemic in 2002 through 2003 that was stopped by a concerted effort that involved strict isolation measures and epidemiologic follow-up (*11*). Although the containment of SARS was a great success in international public health, this and other coronaviruses continue to pose a threat of novel epidemics. The introduction of hCoV-OC43 into humans as a progeny strain of the bovine coronavirus is one example of zoonotic transition of coronaviruses (*12*). Another example is the porcine epidemic diarrhea virus that was introduced into swine in the late 1970s from an unknown source (*13*).

Recently, studies from the People’s Republic of China have identified bats as the most likely source of all coronaviruses (*14*–*18*). Bats constitute ≈20% of all living mammal species (*19*), are distributed on all continents except Antarctica, and occupy diverse ecologic niches in a large range of habitats. Bats exploit a wide dietary diversity, including small vertebrates, nectar, pollen, fruits, blood, and insects (*20*). Almost all bat species live in social groups. The number of bats in such groups ranges from a few up to ≈20 million (*21*), the largest contiguous colonies of mammals on earth (*21*). Typical for all bats is their nocturnal activity and their characteristic roosting behavior during daytime. Roost sites show a great variety that include caves, crevices in rock and tree bark, cavities in tree trunks and branches, foliage, and various human-made structures (*22*). Most bat species regularly use buildings, such as bridges, cellars, mines, wells, and houses, as roosting sites. For this reason, they have contact with humans, possibly enabling virus transmission. Indirect contact by intermediate hosts, such as civet cats and other carnivores, as demonstrated for SARS-CoV, multiply the opportunities of transmission of virus from bats (*23*,*24*).

To predict risks for coronavirus host transition and disease outbreaks, we need to have a deeper understanding of the nature of coronavirus reservoirs, including the association of certain coronaviruses with bat species. Unfortunately, bats are extremely difficult to study, and only few studies of sufficient extent have been conducted. Some recent studies indicate that the same coronavirus may be carried by members of the same species of bats in distant locations. At the same time, different species roosting in the same cave may carry different coronaviruses (*18*,*25*).

Outside China, a very recent study described sequences from group I coronavirus in 2 different North American bat species, *Myotis occultus* and *Eptesicus fuscus* (*26*). Together with our recent observation of anti-coronavirus antibodies in African bats (*27*), this finding suggests that the area of distribution of bat coronaviruses may be considerably larger than currently known. In the Western Palaearctic region (Europe, Middle East, North Africa), 50 bats species from 2 suborders and 6 families are known to exist, and the existence of more cryptic species is likely (*28*). We tested bats prospectively for coronaviruses in the context of an ongoing bat-surveillance program in northern Germany; >300 bats were examined. Coronaviruses were detected, sequenced, and analyzed phylogenetically. Strict species association, as well as a likely nonrecent host transition within bats, was detected. From the locations of capture and physical characteristics of bats (age, sex, lactation, gravidity), implications on transmission and maintenance of bat coronaviruses could be drawn in this study.

## Materials and Methods

### Capture Sites and Sample Collection

**Figure 1:**
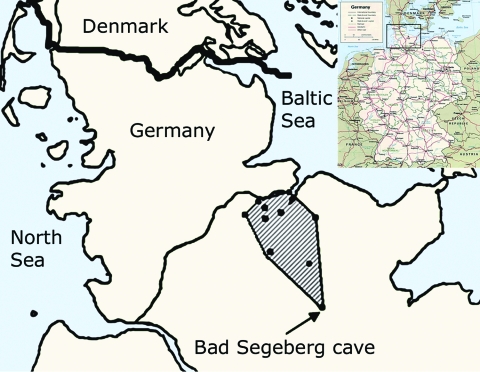
Map of Germany (inset) with enlarged view of northern Germany. The study area is shaded, and dots in the study area indicate sampling sites.

Field work was conducted at 8 sites in a 7,834-km^2^ area northwest of one of the most important winter roosts of bats in central Europe, the Segeberg Cave (10°18′57′′E; 53°56′09′′ N, Figure 1) in the small town of Bad Segeberg (16,000 inhabitants). The town is located in Schleswig Holstein, the northernmost federal state of Germany. The limestone cave shelters >20,000 bats of up to 8 vespertilionid bat species during hibernation. Bats fly up to 115 km from the hibernation site to their summer roosts in the surrounding landscape (*29**,**30*). Bats were caught with mist nets of different length and height in their foraging habitats where bats hunted insects over water; in forest swarming sites, where bats followed courtship behavior; and near maternity roosts, where only adult females and newborns resided, but no adult males. In our region, different species do not share the same maternity sites. Mist nets were checked at intervals of 5 min. Captured bats were freed from nets immediately and put into cotton bags for several minutes to calm down before further investigations started. Species, age category (juvenile, subadult, adult), sex, reproductive status, forearm length, and body mass were determined. Additionally all pond bats (*M. dasycneme*) were marked with aluminum bands for tracking during an ongoing survey and protection program established by the Schleswig-Holstein Federal Ministry of Environment. In 1 case, a radio transmitter (Holohill, Ontario, Canada) was used for bat tracing. While being kept in bags, bats produced fecal pellets that were collected with clean tweezers and spiked into RNAlater RNA stabilization solution (QIAGEN, Hilden, Germany) for sample processing. Duplicate sampling of bats was prevented by marking the toes of captured bats with nail polish upon first catching. Procedures were consistent with national guidelines for the capture, handling, and care of bats.

### Processing and Analysis of Samples

Fecal pellets suspended in RNAlater were homogenized by vortexing. Of the suspensions, 50 μL were introduced into 500 μL of Buffer AVL from the QIAGEN viral RNA minikit and processed further according to the instructions of the manufacturer. Elution volume was 50 μL. Nested reverse transcription–PCR (RT-PCR) was performed exactly as described previously, with primers that provided equally high sensitivity for all coronavirus groups (*31*). Primers targeted a 440-bp fragment of the RNA-dependent RNA polymerase that has been frequently used for phyologenetic comparison of coronaviruses. Because several GenBank entries in this fragment were incomplete, a core region of 334 bp as covered by most GenBank entries was used for analysis. RT-PCR products were sequenced bidirectionally on an ABI 3710 automatic capillary sequencer. Sequences were subjected to nucleic acid alignment by the ClustalW algorithm in the Mega 4 software package (www.megasoftware.net), and analyzed by bootstrapped phylogenetic analysis by using the neighbor-joining algorithm. A nucleic acid distance matrix was also calculated by Mega 4. All analyses on the 334-bp fragment were repeated with the complete 440-bp fragment for validation. All phylogeny and homology results were equivalent, with the exception of a smaller number of complete sequences that were available for the analyses. Results are therefore not shown. All statistical procedures were done with the Statgraphics V 5.1 software package (Manugistics, Dresden, Germany). Sequences from northern German bat coronaviruses can be retrieved from GenBank under accession nos. EU375853–EU375875. Isolation of virus was attempted from diluted RNAlater suspensions, as well as from some fecal pellets suspended separately in phosphate-buffered saline. Vero and CaCo2 cells, as well as primary cell cultures from *Carollia* bat lung and kidney, were used. No virus growth could be confirmed by RT-PCR (data not shown).

## Results

From June 1 to August 31, 2007, bats were caught and classified according to species, sex, age category, gravidity, and lactation status. Bat species were typed by morphologic criteria by experienced bat biologists who had worked in the habitat for several years. A total of 315 bats were sampled (Table 1). Overall prevalence of CoV in all bats was 9.8%.

**Table 1:** Overview of bats tested for coronaviruses (CoV), Germany*

Species	No. bats (positives)	Females	Juveniles/subadults/adults	Gravid	Lactating	Location†
*Myotis bechsteinii*	9 (1)	9	4/0/5	0	2	6
*M. brandtii*	2 (0)	1	0/0/2	1	0	1
*M. dasycneme*	67 (17)	39	33/1/33	0	22	2,† 3,† 5,† 8
*M. daubentonii*	155 (8)	79	17/38/100	5	15	1, 2, 4, 5, 7,† 8†
*Nyctalus noctula*	3 (0)	1	1/0/2	0	1	5
*Pipistrellus nathusii*	22 (2)	13	15/0/7	0	4	1, 3, 5†
*P. pygmaeus*	57 (3)	36	15/0/21	6	10	1, 2, 4, 5†

*Name/ type of habitat /geographic coordinates: location 1, Westensee/ f /9°58′17′′E/54°16′44′′N; 2, Achterwehr/ f /9°57′44′′E/54°18′55′′N; 3, Methorst/ m /9°49′57′′E/54°16′50′′N; 4, Molfsee/ f /10°5′22′′E/54°17′22′′N; 5, Schwentinebrücke/ f /10°17′14′′E/54°16′18′′N; 6, Bornhöved/ m /10°14′14′′E/54°06′04′′; 7, Jägerslust/ s /9°55′26′′E/54°19′40′′N; 8, Projensdorfer Gehölz/ s /10°7′7′′E/54°21′59′′N. †Locations in which CoV-positive bats of this species were found.

Of the 7 species studied, 5 (*M. dasycneme, M. daubentonii, M. bechsteinii, Pipistrellus nathusii, P. pygmaeus*) yielded coronavirus, with detection rates of 5.2% to 25.4% per species. Detection rates varied significantly between bat species, with *M. dasycneme* showing significantly higher rates than any other species (analysis of variance [ANOVA], p<0.0002). Among the *M. dasycneme* bats, detection rates were not equally distributed but correlated significantly with the location in which bats were caught (one-way ANOVA, p<0.013). Similar observations were made for 3 other virus-positive species, where detection was achieved in 2 of 6 (*M. daubentonii*), 1 of 3 (*P. nathusii*), and 1 of 4 (*P. pygmaeus*) sampling locations.

Spillover of bats between colonies was not seen, with one interesting exception. In location 2, one of 7 examined *M. dasycneme* bats was already banded. Its ring number showed that it had spilled over from another area. This bat was the only one yielding coronavirus in location 2. By tagging with a radio transmitter, its maternity roost could be traced to location 3, ≈10 km away. When location 3 was sampled, it yielded 45% positive bats (15 of 38 *M. dasycneme* tested), the highest rate of all locations sampled in the study period.

Factors correlating with coronavirus infection were determined. Analysis was conducted on major physical properties. Approximately half of the bats (56%) were females, 30% of them lactating. Twenty-seven percent were juvenile, 12% subadult, and 61% adult. As shown in Table 2, ANOVA analysis identified that young age and ongoing lactation, but not a particular sex or existing gravidity, correlated significantly positively with coronavirus detection. Among female bats, detection rates were significantly higher in those bats associated with maternity colonies than in those caught in foraging habitats (2-tailed *t* test, p = 0.026) or swarming sites (2-tailed *t* test, p = 0.037). Differences between foraging and swarming sites were not significant (p = 0.609).

**Table 2:** Factors predictive of coronavirus (CoV) detection, Germany

Possible influence factor	Category	% CoV positive	p value*
Age	Juvenile	23.7	0.0015
	Subadult	15.9	
	Adult	8.5	
Sex	Male	17.7	0.39
	Female	14.4	
Lactation	Lactating	22.4	0.021
	Nonlactating	9.7	
Gravidity	Gravid	15.5	0.92
	Not gravid	16.5	

*Indicates level of positive influence on coronavirus detection.

**Figure 2:**
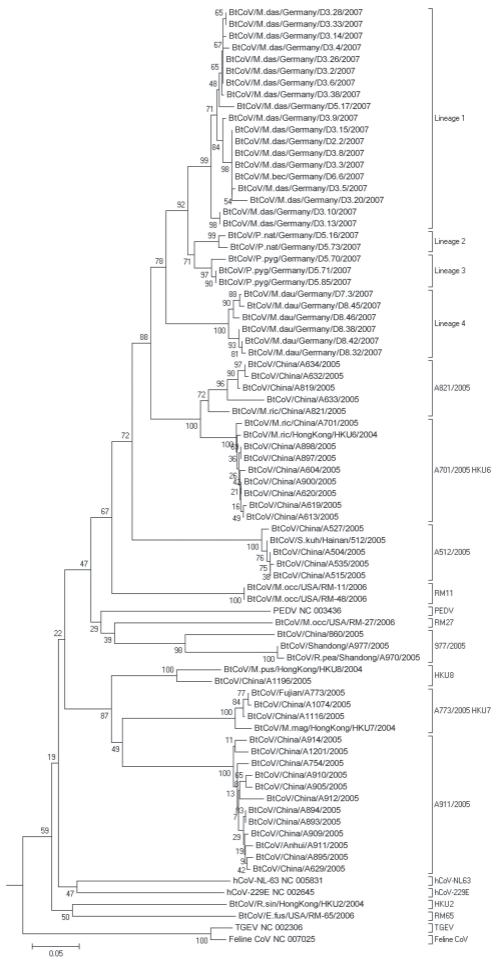
Phlyogenetic analysis of northern German bat coronaviruses (CoV) (lineages 1–4) and related group I CoVs from bats and other mammals. Analyses were conducted in MEGA4 (32), by using the neighbor-joining algorithm with Kimura correction and a bootstrap test of phylogeny. Numbers at nodes denote bootstrap values as percentage of 1,000 repetitive analyses. The phylogeny is rooted with a Leopard CoV, ALC/GX/F230/06 (33). The column on the right shows bat CoV prototype strain names or the designations of type strains of established mammalian CoV species.

Sequences of PCR products from all coronaviruses were determined. As shown in Figure 2, the northern German viruses clustered in 1 large monophyletic clade containing no other previously known virus. In a sister relationship was a clade of viruses from Chinese bats with prototype strains A701/2005, HKU6 and A821/2005 (*18*,*34*). These viruses were all detected in *M. ricketti*, which belongs to the same subgenus (*Leuconoe Boie*) as *M. dasycneme* and uses a similar ecologic niche (*35*–*37*).

Within the Northern German bat-CoV clade, 4 different lineages appeared to be monophyletically associated with certain bat species. Lineage 1 was associated with *M. dasycneme,* lineage 2 with *P. nathusii,* lineage 3 with *P. pygmaeus,* and lineage 4 with *M. daubentonii.* As expected, the coronavirus from the stray bat from location 2 and the viruses from location 3 clustered closely together in lineage 1. Within the same lineage was 1 coronavirus from an *M. dasycneme* bat that had been sampled at a different site and at a different time (location 5), along with *Pipistrellus* bats that carried clearly distinct virus of lineages 2 and 3. Viruses therefore seemed to be more closely associated with bat species than with sampling locations. On the contrary, virus from the only virus-positive *M. bechsteinii* bat, a very rare and almost extinct species, clustered with lineage 1. Virus detection was successfully repeated from the same sample. These findings suggest that spillover of virus from *M. dasycneme* into *M. bechsteinii* might have occurred. In addition, nonrecent host transition of a common ancestor of *Myotis-*associated CoV into *Pipistrellus* is suggested by virus phylogeny for lineage 2 and 3 viruses (Figure 2).

To appreciate the diversity of northern German bat-CoV, a nucleotide distance matrix of 30 major taxa of coronaviruses was set up, including established coronavirus species and novel bat-CoV taxa, as recently defined (Appendix Figure). Lineages 1, 2, and 3 had mutual nucleic acid distances between 6% and 8%. Distance of lineage 4 from the aforementioned was 12%–13%. Distance of northern German lineages 1–4 from the sister clade of Chinese *M. ricketti* CoV was 15%–17%. The Chinese and northern German *Myotis*-associated CoV and their common sister clade, represented by strain A512/2005, were 20%–22% distant. For comparison, among the established species of CoV the lowest degrees of nucleic acid distances were observed between mouse hepatitis virus, human CoV HKU1, and the hCoV-OC43/bovine CoV pair, at 16%–18%. The 2 established lineages within CoV group 2b (SARS-like CoV, bat-SARS CoV HKU3) were 10% distant.

## Discussion

Similar to our previous studies on anti-coronavirus antibodies in African bats and recent findings of bat CoV in North America (*26*,*27*), this study shows that the presence of coronaviruses in bats is not a unique phenomenon in Asia and seems to extend worldwide. The prevalence of coronaviruses in bats in northern Germany was 9.8%, which is in the same range as in studies of similar size from China: Lau et al. found 66 (16%) of 412 bats positive for coronaviruses (*38*), Chu et al. found a prevalence of 15.8% (43/272 bats) (*25*), Woo et al. found 4.2% (13/309 bats) (*34*), and Tang et al. found 6.5% (64/985 bats) (*18*).

To explain how coronaviruses might be transmitted and maintained in bat populations, we have statistically determined factors that influence virus detection. Young bats of both sexes, as well as lactating bats, but not gravid bats, were significantly more likely to carry coronaviruses. The virus could be transmitted between young bats and mothers in maternity colonies, rather than circulating year-round at equal levels in the population. Indeed, female bats captured near maternity colonies showed significantly higher virus detection rates than those captured in foraging or swarming sites. Our bats reside in maternity colonies during early summer months; after young bats are born, male bats avoid maternity colonies. One could propose a scenario in which the young provide a susceptible population, amplifying the virus and transmitting it to adult females in maternity colonies. Comparable to many respiratory and enteric virus infections in humans, adults would replicate virus less efficiently than the young because they have at least partial immune protection because of infection earlier in life. This would explain lower detection rates in adult bats. For confirmation, the immune status of young and adult bats against homologous coronaviruses would need to be studied. However, we cannot take blood from our bats without harming them because of their small size; bats in Germany are strictly protected.

Similar to studies conducted in China (*18*,*25*), viruses might be associated with bat species. The same virus was never detected in different species that occurred simultaneously in the same location. For example, *M. daubentonii* and *M. dasycneme* both occurred in locations 2 and 8, but only 1 species per location yielded (different) virus; location 5 harbored all 4 virus-carrying species in 1 place. Three of 4 *Myotis* species yielded viruses (Table 1), and these belonged to 3 different lineages. On the other hand, the same virus lineage was found in remote colonies of the same bat species (compare *M. dasycneme* and *M. daubentonii* in Table 1).

On the local scale of our study, it was difficult to determine whether strict species association or limited local transmission may be responsible for the observed associations is difficult to determine. An influence of local transmission cannot be excluded, considering the hypothesis that virus is likely transmitted in maternity colonies. Groups of bats from the same maternity colony stay together throughout life, and bats of the investigated species never mix in such colonies.

On a larger scale, however, a group I coronavirus hosted by palaearctic (or Old World) *Myotis* bats, including *M. dasycneme* and *daubentonii* in Germany and *M. ricketti* in China (*18*,*34*), might exist. Earlier studies (*18*) used a threshold of 20% nucleic acid distance in our target gene to define a new species of bat coronavirus. By using these criteria, the palearctic *Myotis* virus would form a distinct coronavirus species with German and Chinese subspecies. Even though such a classification is preliminary and does not take other aspects of coronavirus classification into account, it would be supported by host biology. *Myotis* bats do not migrate, but habitats of different *Myotis* species continuously overlap from China throughout Asia into Europe. *M. ricketti,* which harbors the Chinese sister clade of our coronaviruses, belongs to the same subgenus (*Leuconoe Boie*) as *M. dasycneme* and uses a similar ecologic niche (*35*–*37*). A continuous virus population might thus coexist with a continuous palearctic *Myotis* population. As a conclusive extension of this hypothesis, the recently described North American bat coronaviruses RM11 and RM48 from *M. occultus* were more closely related to our viruses than the RM65 strain from an unrelated *Eptesicus fuscus* bat (*26*).

Finally, the virus observed in 2 different *Pipistrellus* species would likely have resulted from a host switch of *Myotis* virus; *Pipistrellus* is not closely related to *Myotis* spp. (*28*). As predicted very recently, coronaviruses may not only be prone to accidental, infrequent host switch between mammals, but may jump from 1 host species to another within the bat reservoir (*39*). Our study supports this notion and suggests that host transition within geographically closely associated, but genetically distinct, bats may have occurred. Because all related viruses in Europe and China were associated with *Myotis*, the direction of transition was probably from *Myotis* spp. into *Pipistrellus* spp., where virus then would have diversified further. This hypothesis is also suggested by relatively long internal branch lengths on the third level of bifurcation (counting from the basal node of the German *Myotis* clade) that separates both *Pipistrellus* virus lineages. This in turn could be a correlate of independent adaptations to *P. nathusii* and *P. pygmaeus*, respectively, after host transition.

Should these initial observations be confirmed in future studies, implications on infection control and prevention of zoonotic outbreaks would be considerable. Targeted eradication of bats is technically impossible and ecologically detrimental. Systematic intervention in the ability of bats to carry coronaviruses might be a realistic, but remote scenario. Further research into the association of coronaviruses with natural hosts is necessary to understand their maintenance patterns and zoonotic potential.

## Supplementary Material

Appendix Figure Nucleic acid distances in a 334-bp fragment of ORF1b retrievable from most coronaviruses. Pairwise nucleic acid distances in the 334-bp core fragment of ORF1 that has been completely entered in GenBank for most coronaviruses from a 440-bp amplification product. Distances between prototype bat coronaviruses or type strains of established mammalian coronavirus species of groups I and II are shown. Names of type strains or bat coronavirus prototype strains are shown in the left column. Distance values ≤0.1 are underlined.
