# Supplementary figures and images for: Detection and Prevalence Patterns of Group I Coronaviruses in Bats, Northern Germany
Source: Emerg Infect Dis. 2008 Apr;14(4):626–31. doi: 10.3201/eid1404.071439 (PMC2570906; doi:10.3201/eid1404.071439)

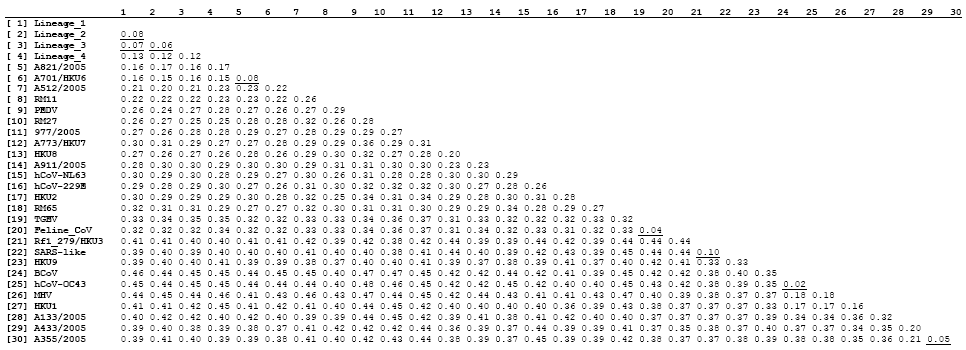

Supplement: Appendix Figure — Nucleic acid distances in a 334-bp fragment of ORF1b retrievable from most coronaviruses. Pairwise nucleic acid distances in the 334-bp core fragment of ORF1 that has been completely entered in GenBank for most coronaviruses from a 440-bp amplification product. Distances between prototype bat coronaviruses or type strains of established mammalian coronavirus species of groups I and II are shown. Names of type strains or bat coronavirus prototype strains are shown in the left column. Distance values ≤0.1 are underlined. [file 07-1439_app.gif]
